# Natural genetic variation in calcium sensor genes as a novel resource for abiotic stress tolerance in crops

**DOI:** 10.3389/fpls.2026.1747177

**Published:** 2026-03-09

**Authors:** Abdul Kadir Issah, Yu Wang, Samuel Azupio, Hongmei Zhang, Jing Chu, Warahama Sayibu, Qing Xie, Xingyu Jiang

**Affiliations:** 1 National Center of Technology Innovation for Saline-Alkali Tolerant Rice, College of Coastal Agricultural Sciences, Guangdong Ocean University, Zhanjiang, China; 2 Council for Scientific and Industrial Research, Oil Palm Research Institute, Kade, Ghana; 3 Faculty of Agriculture, Food and Consumer Sciences, University for Development Studies, Tamale, Ghana

**Keywords:** abiotic stress tolerance, calcium signaling, climate-resilient crops, crop wild relatives, genome editing, natural genetic variation

## Abstract

Abiotic stress represents a significant and increasing challenge to global crop productivity and food security. Calcium (Ca^2+^) signaling, initiated by specific “Ca^2+^ signatures” and interpreted by sensor proteins such as calcium-dependent protein kinases (CDPKs/CPKs) and the CBL-CIPK network, functions as a key regulator of plant adaptive responses. However, contemporary elite cultivars exhibit a reduced genetic base, having forfeited numerous resilient alleles present in wild relatives and landraces during intensive, yield-focused breeding. This review synthesizes evidence demonstrating that natural genetic variation within these calcium sensor genes significantly influences key agronomic traits, including ion homeostasis, stomatal regulation, and water-use efficiency. We then evaluate​ the effectiveness of integrated genomic approaches, such as pan-genomics, genome-wide association studies (GWAS), and CRISPR-Cas9 genome editing, for systematically identifying and validating these beneficial alleles. Finally, we propose a translational roadmap for the targeted introgression of enhanced calcium sensor variants into modern germplasm. This work provides a strategic framework for developing a new generation of climate-resilient crops, offering a pathway to safeguard global food systems against increasingly erratic environmental conditions.

## 1 Introduction

Global food security is increasingly threatened by the mounting pressure of abiotic stresses. Drought, salinity, temperature extremes, and nutrient deficiency remain primary constraints on global agricultural productivity (Heino et al., 2023; Tarolli et al., 2024). Climate change is exacerbating these threats by altering their frequency, intensity, and geographic distribution. As a result, developing stress-resistant crops has become a critical imperative for sustainable agriculture and long-term food security.

Unlike motile animals, plants are sessile organisms that must withstand adverse conditions by activating intrinsic physiological and molecular responses (Saharan et al., 2022). The perception of stress at the cellular level triggers a complex signaling cascade. This cascade is initiated at the plasma membrane, where the activation of Receptor-Like Kinases (RLKs) and ion channels leads to global transcriptional reprogramming, metabolic changes, and physiological remodeling (Shi et al., 2023; Zuo et al., 2025). Among the earliest and most rapid cellular responses are alterations in cytosolic calcium (Ca²^+^) homeostasis (Negi et al., 2023). Following stimuli such as osmotic shock, high salinity, or cold treatment, the cytosolic Ca²^+^ concentration increases within seconds (Kudla et al., 2018). These stimulus-specific changes, termed “Ca²^+^ signatures, “ vary in amplitude, frequency, duration, and subcellular location, thereby encoding precise information about the nature and severity of the stress (Schmöckel et al., 2015).

The information within these Ca²^+^ signatures is decoded by an array of sensor proteins that act as primary transducers, linking the Ca²^+^ signal to downstream biological outputs (Ranty et al., 2016). Key sensor families include calcium-dependent protein kinases (CPKs/CDPKs), calcineurin B-like proteins (CBLs) and their interacting kinases (CIPKs), calmodulins (CaMs), and calmodulin-like (CML) proteins. For instance, ​ CDPKs are “sensor-responders” that contain both Ca²^+^-binding EF-hand domains and a serine/threonine kinase domain, enabling them to directly phosphorylate targets such as transcription factors and ion channels in response to Ca²^+^ fluctuations (Bredow et al., 2021; Li et al., 2024). Similarly, the CBL-CIPK network, exemplified by the Salt Overly Sensitive (SOS) pathway, regulates ion transporters, such as SOS1, to maintain ion homeostasis under saline stress (Tagliani et al., 2020; Kaya et al., 2024). In contrast, ​ CaM and CML proteins are “sensor-relays” that, upon Ca²^+^ binding, modulate the activity of a diverse array of target proteins but lack enzymatic activity themselves (Cai et al., 2022). Collectively, these sensors form a sophisticated toolkit that enables plants to perceive environmental cues and mount appropriate adaptive responses.

While the molecular functions of calcium signalling have been extensively investigated, research has largely focused on the model dicot *Arabidopsis thaliana*. This model-centric approach, while essential for establishing general principles, neglects the genetic diversity and species-specific adaptations of key cereals, including rice, wheat, and maize. Furthermore, contemporary breeding practices aimed at enhancing yield and uniformity have resulted in a genetic bottleneck, unintentionally eliminating stress-tolerant alleles from landraces and wild relatives (Allaby et al., 2018). Thus, while calcium mobilisation is a conserved pathway, the inherent genetic variation within its sensor network is largely an unexplored resource for breeding (Dong et al., 2022). This unexplored variation presents a significant opportunity to improve crop resilience.

Emerging evidence supports the functional relevance of this variation. For instance, allelic diversity in rice CBL and CIPK genes modulates salt tolerance through differential kinetics of ion transport (Chen et al., 2021). Natural alleles of specific CDPK genes in Arabidopsis and tomato are associated with variations in drought-induced stomatal closure and water-use efficiency (Bhaskara et al., 2022). These examples illustrate how subtle sequence changes can fine-tune signaling pathways and drive complex phenotypic outcomes (Puli et al., 2024; Jochim et al., 2025). However, the systematic mining and functional characterization of this natural variation across diverse germplasm collections remain in their infancy.

Here, we propose a critical strategic shift in research focus to translate mechanistic insights from model systems into tools for crop improvement. Recent technological advances have rendered this goal achievable. High-throughput phenotyping (HTP) technologies enable the efficient determination of stress-resilience traits in large populations.​ Genomic tools such as genome-wide association studies (GWAS) and quantitative trait locus (QTL) mapping link genetic variation to phenotypic traits, identifying candidate loci (Korte and Farlow, 2013). The pan-genome concept, which encompasses the entire set of genes within a species, reveals the presence-absence variations (PAVs) and structural variants (SVs) that are missed by a single reference genome, documenting a richer tapestry of genetic diversity (Chapman et al., 2022). Functional genomic tools such as CRISPR-Cas9 facilitate the transition from correlation to causation by validating gene-trait relationships and enabling the precise introduction of beneficial alleles into elite cultivars. The convergence of these technologies enables the systematic mining and utilisation of the untapped variation in calcium signalling genes. We propose a coordinated research strategy that integrates genomic and precision breeding tools to develop a new generation of climate-resilient crops, thus safeguarding global food systems from increasingly erratic environmental conditions.

## 2 Calcium signaling in plants

Calcium (Ca^2+^) is a multifunctional cation that serves as both an essential structural nutrient and a ubiquitous intracellular secondary messenger in plants. Its signaling function is central to mediating growth, development, and, crucially, acclimatization to unfavorable environmental conditions (Ren et al., 2023). This section outlines the fundamental principles of the Ca^2+^ signaling pathway, from stimulus perception to the activation of adaptive responses.

### 2.1 The Ca^2+^ signature: a encoded message for stress perception

The initial perception of abiotic stresses, such as drought, salinity, and temperature shock, triggers one of the earliest cellular responses: a transient elevation of cytosolic Ca^2+^ levels (Thor, 2019; Weng et al., 2022). This is not a generic alarm signal but a specific “Ca^2+^ signature” characterized by spatiotemporal variations in amplitude, frequency, duration, and subcellular location (Resentini et al., 2021). For instance, the Ca^2+^ influx from the vacuole activated by cold can be distinguished from the signature elicited by osmotic stress, enabling the plant to discriminate between different environmental threats (Yuan et al., 2018). These complex Ca^2+^ fingerprints are generated by the coordinated activity of an array of Ca^2+^-permeable channels and transporters located at the plasma membrane and in internal organelles, making Ca^2+^ signaling a highly sophisticated process (Casas and Dickson, 2024). Key channels involved include Cyclic Nucleotide-Gated Cation Channels (CNGCs), Glutamate Receptor-Like (GLR) channels, and Two-Pore Channels (TPCs), which convert specific extracellular stimuli into unique intracellular Ca^2+^ signatures that encode information for downstream cellular processes (Jha et al., 2016; Toyota et al., 2018; Jin et al., 2020).

### 2.2 Decoding the signature: families of calcium sensor proteins

**Figure 1:**
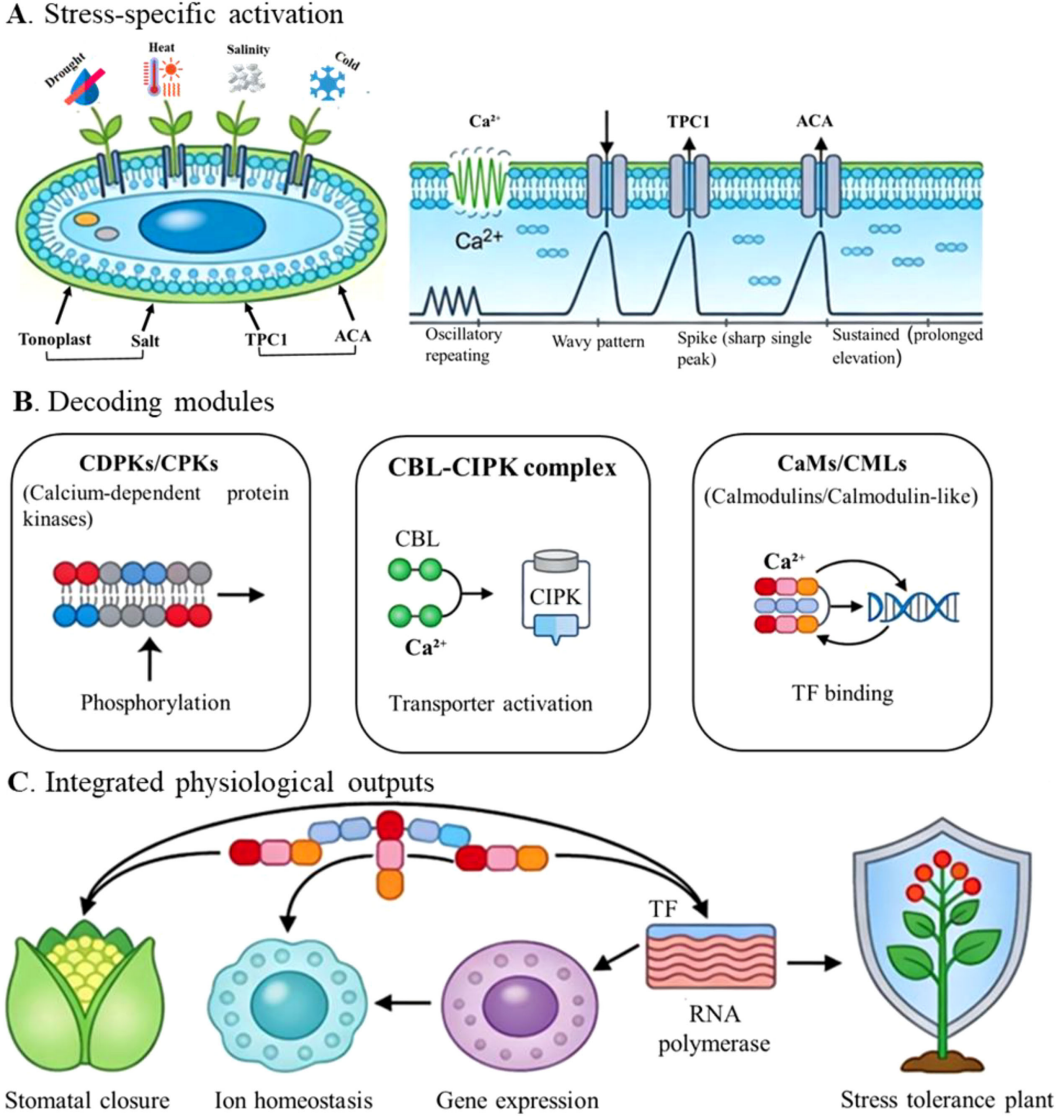
The calcium signaling toolkit: from stress perception to adaptive response. Schematic representation of calcium-mediated signaling in plant responses to abiotic stress. **(A)** Stress-specific activation of cytosolic Ca^2+^ signatures in response to drought, heat, salinity, and cold generates distinct temporal Ca^2+^ patterns, including oscillatory, wavy, spike-like, and sustained elevations, via Ca^2+^-permeable channels such as TPC1 at the tonoplast and Ca^2+^-ATPases (ACA) at the plasma membrane and endomembranes. **(B)** Major Ca^2+^-decoding modules translate these signatures into biochemical responses: calcium-dependent protein kinases (CDPKs/CPKs) phosphorylate downstream targets; calcineurin B-like (CBL) proteins interact with CBL-interacting protein kinases (CIPKs) to regulate ion transporters; and calmodulins/calmodulin-like proteins (CaMs/CMLs) bind Ca^2+^ and modulate transcription factors (TFs). **(C)** Integration of these signaling pathways results in key physiological outputs, including stomatal closure, ion homeostasis, and reprogramming of gene expression through TF and RNA polymerase–mediated transcription, ultimately enhancing overall plant stress tolerance.

A complex set of sensor proteins interprets the information encoded within the Ca^2+^ signature. These molecular translators convert the rapid chemical signal into a sustained physiological response (Plasencia et al., 2021). The position and role of these primary sensor families within the broader calcium signaling pathway are illustrated in **Figure 1**.​ The primary sensor families can be categorized into three main groups:

Calcium-dependent protein kinases (CDPKs/CPKs):​ These are “sensor-responders” characterized by a unique structure that combines Ca^2+^-binding EF-hand domains and a serine/threonine kinase domain on a single polypeptide. This allows CDPKs to directly sense changes in cytosolic Ca^2+^ and immediately phosphorylate downstream targets, such as transcription factors, ion channels, and metabolic enzymes (Dekomah et al., 2022; Wernimont et al., 2010).

The CBL-CIPK Network:​ This two-component system acts as a central relay. Ca^2+^ binding induces conformational changes in Calcineurin B-Like (CBL) proteins, which then recruit and activate specific CBL-Interacting Protein Kinases (CIPKs). The activated CIPKs phosphorylate target proteins to regulate specific pathways. A well-characterized example is the Salt Overly Sensitive (SOS) pathway, where the CBL4-CIPK24 complex activates the SOS1 Na^+^/H^+^ antiporter to promote sodium efflux and ion homeostasis under saline stress (Tang et al., 2020; Sanyal et al., 2020).

Calmodulin and Calmodulin-like proteins (CaMs/CMLs):​ These small, multifunctional “sensor-relays” do not possess enzymatic activity themselves. Upon Ca^2+^-binding, they undergo a conformational change that enables them to bind and modulate the activity of a diverse array of target proteins, including metabolic enzymes and transcription factors (Charoenwattanasatien et al., 2020; Yang and Tsai, 2021).

### 2.3 Specificity, integration, and physiological outcomes

The robustness of calcium signaling stems from its dual capacity for specificity and integration. Specificity is achieved through stimulus-specific Ca^2+^ signatures and the selective activation of sensor proteins at distinct intracellular sites (Yuan et al., 2017). Simultaneously, the pathway acts as a central integration hub, engaging in extensive crosstalk with other major signaling cascades, such as reactive oxygen species (ROS) signaling, MAPK pathways, and phytohormone networks, particularly abscisic acid (ABA) (Jagodzik et al., 2018).

**Figure 2:**
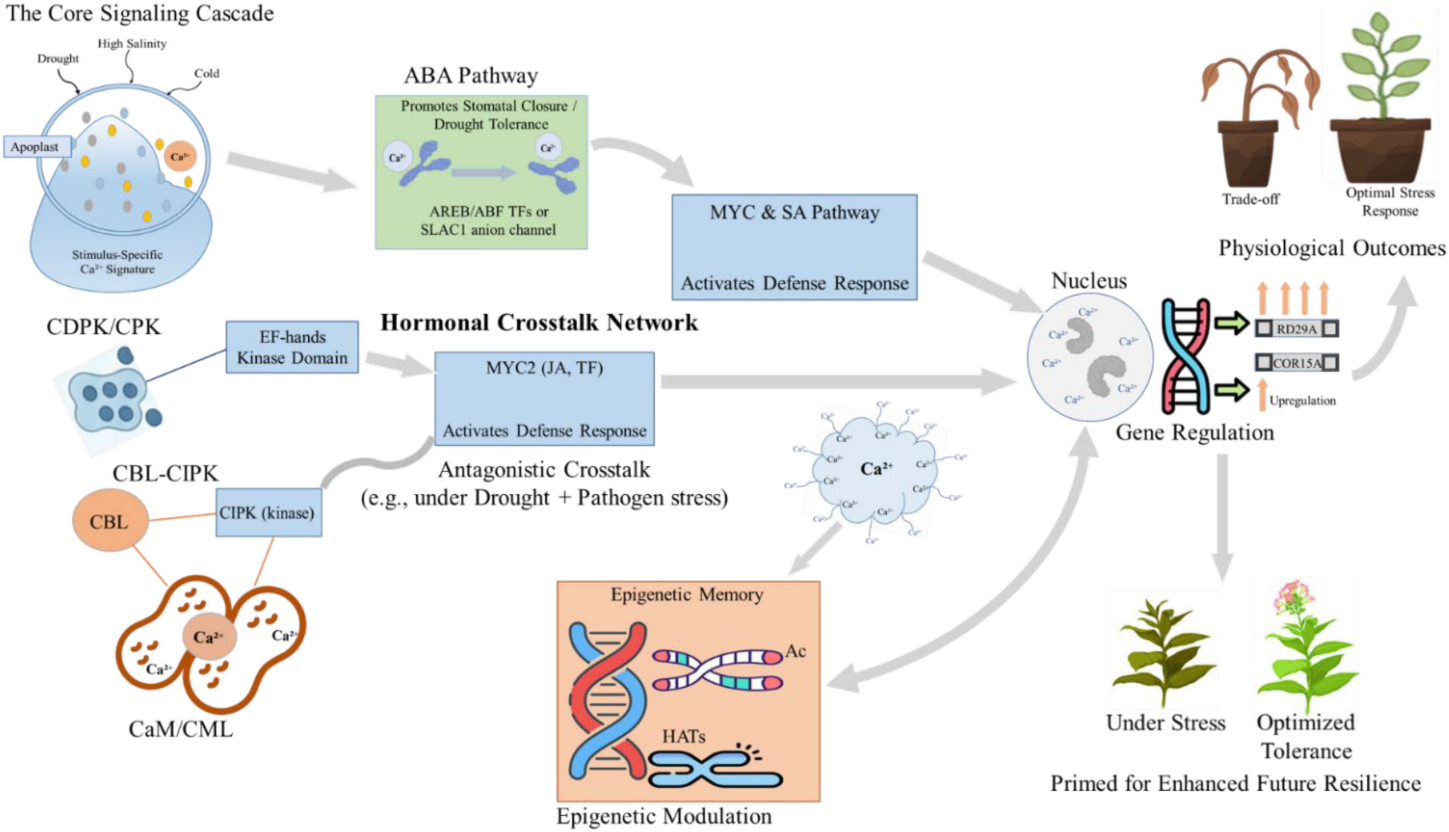
Calcium signaling integrates phytohormone pathways and transcriptional networks to orchestrate abiotic stress responses. Model of Ca^2+^-mediated integration of hormonal signaling, gene regulation, and epigenetic memory in plant abiotic stress responses. Stimulus-specific Ca^2+^ signatures generated under drought, high salinity, or cold are decoded by Ca^2+^-dependent modules such as CDPK/CPK, CBL–CIPK, and CaM/CML complexes, which feed into an interconnected hormonal crosstalk network. In this network, Ca^2+^-regulated components modulate abscisic acid (ABA) signaling to promote stomatal closure and drought tolerance, and interact with MYC and salicylic acid (SA)-dependent pathways to activate defense-related transcription factors (e.g., MYC2). The integrated Ca^2+^ and hormonal signals converge in the nucleus to reprogram gene expression, including upregulation of canonical stress-responsive genes such as RD29A and COR15A, and are further stabilized by epigenetic modulation through histone acetyltransferases (HATs) and associated chromatin changes that establish stress memory. These combined processes determine physiological outcomes ranging from growth–defense trade-offs under stress to optimized tolerance and enhanced resilience upon subsequent stress exposure.

The effectiveness of calcium signalling is enhanced by its significant interaction with phytohormone networks, creating integrated regulatory modules that coordinate stress responses. This intricate bidirectional crosstalk, in which calcium signals both affect and are affected by hormonal pathways to refine transcriptional responses, is depicted in **Figure 2**. Calcium ions often act as secondary messengers in hormone signalling. For example, the stomatal closure induced by abscisic acid (ABA) is mediated by Ca^2+^ signatures that activate particular CDPKs and CBL-CIPK complexes. Calcium signals can influence hormone biosynthesis and signaling pathways, leading to intricate feedback loops. A recent overview by Seth et al. (2025) emphasises that this bidirectional crosstalk is essential for customising plant responses to concurrent abiotic and biotic stresses. Calcium-dependent protein kinases (CDPKs/CPKs) phosphorylate critical components in the abscisic acid (ABA), jasmonic acid (JA), and salicylic acid (SA) signalling pathways, thus influencing the intensity and specificity of hormonal responses. This integration guarantees that the calcium signature is understood within a coordinated hormonal framework, facilitating the precise adjustment of physiological outcomes, including growth-defense trade-offs under simultaneous stresses.

For example, during drought stress, ABA accumulation triggers a cytosolic Ca^2+^ increase, which is then transduced through specific CDPKs and the CBL-CIPK module to regulate stomatal closure, activate antioxidant defenses, and stimulate the production of protective osmolytes (Liu et al., 2022b). Similarly, under cold stress, Ca^2+^ signals interact with the C-repeat Binding Factor (CBF) transcriptional cascade to induce cold-responsive genes and enhance freezing tolerance (Hwarari et al., 2022; Yuan et al., 2018). The integrative capacity is essential in combined stress scenarios, such as simultaneous drought and heat or abiotic-biotic stress combinations. In these situations, calcium signatures must navigate conflicting response pathways, resulting in distinct transcriptional and physiological outcomes that cannot be anticipated from single-stress studies (Jiang et al., 2025).

In addition to immediate signalling events, calcium transients can induce long-term transcriptional memory via epigenetic modifications, thereby contributing a significant regulatory layer to the stress response. Variations in cytosolic Ca^2+^ concentrations can affect the function of enzymes involved in DNA methylation and histone modifications, including acetyltransferases, deacetylases, and methyltransferases. Epigenetic modifications can establish stable “epialleles” of calcium sensor genes, potentially enhancing their expression in response to future stressors. A recent study on tomatoes indicated that drought-induced Ca^2+^ spikes are associated with specific histone modifications at the promoters of key transcription factors, which regulate the expression of stress-responsive genes and facilitate acclimation (Ge et al., 2025). This interaction indicates that Ca^2+^ signals not only trigger immediate responses but also have the potential to epigenetically reprogram the plant for improved resilience in the future.

Beyond stress adaptation, calcium signaling, particularly through CaMs/CMLs, is integral to normal plant development, regulating processes such as cell polarity, root system architecture, and the circadian clock (Jang et al., 2024). This dual role in both development and stress responses underscores the critical importance of Ca^2+^ ion homeostasis for plant survival (Huang et al., 2022).

The intricate system of calcium signatures, channels, and sensor proteins constitutes a vital network that allows plants to detect and respond appropriately to abiotic stress, as demonstrated in **Figures 1** and **2** above. This foundational understanding prompts an important inquiry: how does natural genetic variation within this conserved signalling toolkit influence the diversity of stress tolerance seen among plant species?

## 3 Natural variation in calcium signaling genes: from alleles to adaptive traits

### 3.1 The spectrum of genetic variation in calcium signaling genes

Natural genetic variation provides the fundamental substrate for phenotypic diversity and adaptive evolution. This variation manifests in many forms, each with the potential to fine-tune how plants perceive and transduce environmental cues via calcium (Ca^2+^) signaling networks. These alterations range from single-nucleotide changes to large-scale structural variations, all contributing to the functional diversity of Ca^2+^ sensors, decoders, and transporters.

Single-nucleotide polymorphisms (SNPs) are the most prevalent form of genetic variation. Their genomic location determines their functional impact. Non-synonymous SNPs​ within exonic regions can lead to amino acid substitutions that alter critical protein properties, such as the Ca^2+^-binding affinity of sensors (e.g., CaMs, CBLs), the enzymatic activity of kinases like CDPKs and CIPKs, or the gating properties of Ca^2+^ channels (Huang et al., 2023). Even synonymous SNPs, which do not change the amino acid sequence, can influence mRNA stability, translation efficiency, and codon usage bias (Li and Chen, 2023). Furthermore, regulatory SNPs in promoter or enhancer regions can modulate the timing, tissue specificity, and amplitude of gene expression. For example, genome-wide association studies (GWAS) have linked regulatory polymorphisms to calcium deposition and drought tolerance in finger millet (Gebreyohannes et al., 2025).

Beyond SNPs, insertions and deletions (indels) represent another major source of variation. Frameshift indels can lead to a complete loss of function by disrupting the reading frame of key calcium signaling genes. Even non-frameshift indels can affect protein function if they occur within critical domains, such as the EF-hand motifs essential for Ca^2+^ binding. On a larger scale, structural variations (SVs), including duplications, deletions, inversions, and translocations, can dramatically reshape genome architecture (Lalonde et al., 2017). Gene duplication events, common in large families such as the CIPKs and CMLs, can lead to functional redundancy or neofunctionalization, enabling specialized stress responses (Kawasaki and Kretsinger, 2017). Copy number variations (CNVs) can also directly influence gene dosage and protein abundance (Medeiros et al., 2024).

**Figure 3:**
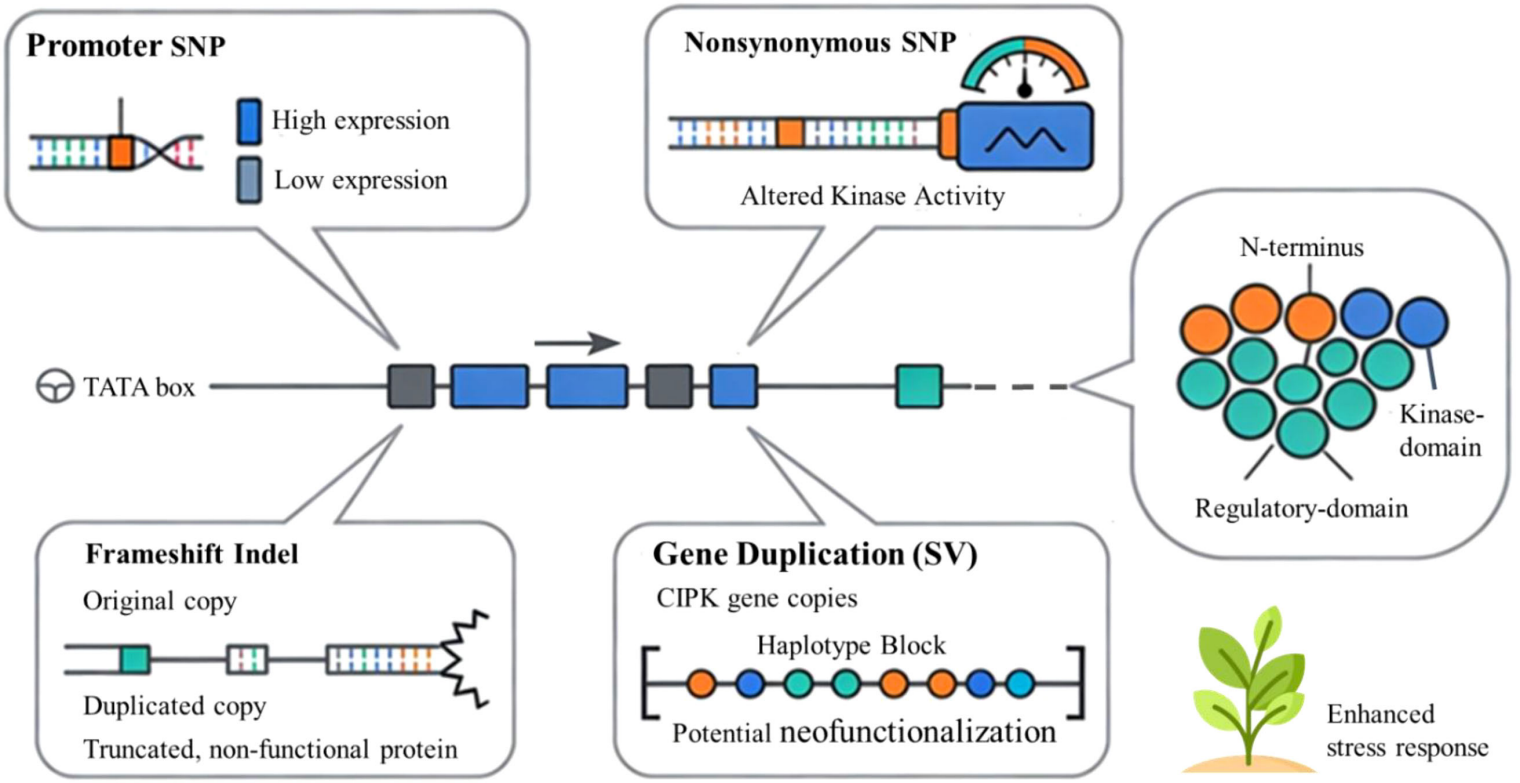
Spectrum of natural genetic variation in calcium sensor genes. Genetic variation shaping CIPK gene function and plant stress responses. Schematic representation of a CIPK gene illustrating how distinct classes of genetic variants can modulate gene expression, protein structure, and signaling capacity. Promoter single-nucleotide polymorphisms (SNPs) influence transcriptional output, resulting in high or low CIPK expression, whereas nonsynonymous coding SNPs alter amino acid sequence and kinase activity. Frameshift-inducing insertions/deletions (indels) in duplicated copies can generate truncated, non-functional proteins, in contrast to intact original alleles, while larger structural variants such as gene duplications expand CIPK copy number within haplotype blocks and enable potential neofunctionalization. Together, these regulatory and coding variants reshape N-terminal, regulatory, and kinase-domain architecture and can ultimately enhance plant stress response capacity.

The functional unit of inheritance is typically the haplotype, which consists of a combination of co-inherited alleles located on a chromosomal segment. Haplotype-based analysis often provides greater insight than single-SNP methods, as it encompasses epistatic interactions among closely linked polymorphic sites that jointly affect gene expression and protein function. A particular haplotype within a CDPK gene may provide a selective advantage under stress, which could be overlooked when examining individual SNPs in isolation (Forsberg et al., 2025). The functional impact of genetic variation in calcium signaling components arises from a hierarchy of changes, ranging from single-nucleotide alterations that affect protein function to complex haplotypes that influence adaptive phenotypes, as shown in **Figure 3**. This section examines particular case studies of variation within major sensor families.

### 3.2 Case studies of variation in major sensor families

The functional implications of genetic variation are illustrated by natural alleles found in core calcium sensor families across various species, connecting sequence diversity to adaptive phenotypic results (**Table 1**).

**Table 1:** Examples of natural allelic variation in calcium sensor genes conferring abiotic stress tolerance.

Gene (Species)	Sensor family	Type of variation	Stress phenotype	Proposed mechanism/effect	Reference
*AtCIPK6* (*Arabidopsis*)	CDPK/CPK	Natural alleles	Drought, Salt Tolerance	ABA-dependent osmotic adjustment	(Chen et al., 2013)
*OsCPK12* (Rice)	CDPK/CPK	Allelic variation	Salt Tolerance	Promotes tolerance through ABA signaling and ROS scavenging	(Asano et al., 2012)
*OsCPK17, OsCPK21* (Rice)	CDPK/CPK	Specific haplotypes	Cold and Salt Tolerance	Associated with cold and salt tolerance, respectively	(Asano et al., 2011; Almadanim et al., 2017)
*ZmCPK4* (Maize)	CDPK/CPK	Natural variation	Drought Tolerance	Modulates stomatal conductance and ABA signaling pathway	(Jiang et al., 2013)
*OsCIPK24* (Rice)	CBL-CIPK	Natural alleles	Salt Tolerance	Critical for activating SOS1 Na^+^/H^+^ antiporter in the SOS pathway.	(Kanwar et al., 2014)
*OsCIPK12* (Rice)	CBL-CIPK	Natural alleles	Drought Tolerance	ABA-dependent regulation of stomatal closure and antioxidant defense	(Todaka et al., 2015)
*HvCBL4* (Barley)	CBL-CIPK	Natural variation	Drought Tolerance	Associated with maintained yield under water-limited conditions	(Cai et al., 2022)
*CaCPK1* (Chickpea)	CDPK/CPK	Expression QTL (eQTL)	Drought, Salt Tolerance	Positive regulator of ABA-dependent stomatal closure and ROS scavenging	(Deepika et al., 2022)
*GmCIPK2* (Soybean)	CBL-CIPK	Overexpression	Salt Tolerance	Enhances expression of ion transporter genes and ROS detoxification enzymes	(Chakraborty et al., 2023)
*SiCIPK10* (Tomato)	CBL-CIPK	Natural variants	Drought Tolerance	Regulates ROS scavenging and photosynthetic protection	(Ge et al., 2025)

This table provides representative examples of how natural genetic variation in calcium sensor genes influences abiotic stress tolerance across different crop species. The listed genes and mechanisms are discussed in the narrative of Section 3.2.

#### 3.2.1 Variation in the CDPK/CPK family

Natural variation within the Calcium-dependent protein kinase (CDPKs/CPKs) family significantly influences abiotic stress responses. In *Arabidopsis thaliana*, natural alleles of *AtCPK6* are associated with enhanced drought and salt-stress tolerance through ABA-dependent osmotic adjustment (Chen et al., 2013). Conversely, loss-of-function alleles of *AtCPK8* impair stomatal regulation and water-use efficiency (Bhaskara et al., 2022). In rice, allelic variation in *OsCPK12* promotes salt tolerance through ABA signaling and reactive oxygen species (ROS) scavenging (Asano et al., 2012). Meanwhile, specific haplotypes of *OsCPK17* and *OsCPK21* are linked to cold and salt tolerance, respectively (Asano et al., 2011; Almadanim et al., 2017). Similarly, in maize, natural variation in *ZmCPK4* modulates stomatal conductance and the ABA signaling pathway under drought stress (Jiang et al., 2013). These examples underscore the role of CDPK allelic diversity in fine-tuning stress-specific signaling pathways.

#### 3.2.2 Variation in the CBL-CIPK network

The CBL-CIPK network is a key regulator of ion homeostasis, and its natural variation is crucial for salinity tolerance. The most prominent example is the Salt Overly Sensitive (SOS) pathway in rice. Natural alleles of OsCBL4 (SOS3) and its interacting kinase *OsCIPK24* (SOS2) are critical for activating the SOS1 Na^+^/H^+^ antiporter, which mediates sodium extrusion from cells (Kanwar et al., 2014). Beyond the SOS pathway, variation in other modules, such as the OsCBL1-O*sCIPK23* pair, modulates potassium homeostasis under saline and low-potassium conditions (Li et al., 2014). Association studies in wheat and barley have also identified SNPs in CBL and CIPK genes linked to differential drought, salt, and heat-stress responses (Schierenbeck et al., 2023; Slawin et al., 2024), highlighting the conserved importance of variation in this network.

#### 3.2.3 Variation in calmodulin and other sensors

Natural variation also exists within the large families of Calmodulin (CaM) and Calmodulin-Like (CML) proteins, which act as Ca^2+^sensor relays. While less explored in an abiotic stress context, allelic differences in these genes can influence the interaction with and activation of diverse target proteins, such as metabolic enzymes and transcription factors (Yang and Tsai, 2021). Furthermore, variation in genes encoding Ca^2+^ channels themselves, such as Cyclic Nucleotide-Gated Channels (CNGCs) and Glutamate Receptor-Like (GLR) proteins, can alter the influx of Ca^2+^ and the very initiation of the stress signature (Jha et al., 2016; Toyota et al., 2018). These case studies establish a direct connection between sequence variation in CDPK, CBL-CIPK, and related genes and quantifiable physiological outcomes, thereby offering a robust basis for candidate alleles. The subsequent essential step involves systematically extracting these alleles from the extensive genetic resources provided by wild relatives and landraces, as elaborated in the following section.

#### 3.2.4 Variation in legume calcium sensors and symbiotic signaling

While major cereals have been a primary focus, understanding calcium signaling variation in legume crops is equally critical due to their high susceptibility to salinity, waterlogging, and nutrient deficiencies, as well as their unique role in sustainable agriculture through nitrogen fixation. Abiotic stress tolerance in legumes is intricately linked to their calcium signaling networks. For instance, in chickpea (*Cicer arietinum*), calcium-dependent protein kinases, such as CaCPK1, are positive regulators of ABA-dependent stomatal closure and ROS scavenging under drought and salt stress (Deepika et al., 2022). Similarly, in soybean (*Glycine max*), natural variation in *GmCIPK2* influences salt tolerance by enhancing the expression of ion transporters and antioxidant defense enzymes (Chakraborty et al., 2023). Furthermore, pan-genomic analyses in soybean have revealed significant presence-absence variation in calcium sensor genes, highlighting an unexplored reservoir of alleles that may contribute to local adaptation (Wicaksono and Buaboocha, 2024).

A particularly distinctive aspect of calcium signaling in legumes is its central role in establishing root nodule symbiosis with nitrogen-fixing rhizobia. This process involves highly orchestrated nuclear-associated Ca^2+^ oscillations (“calcium spiking”), which are decoded by a specific calcium-calmodulin-dependent protein kinase (CCaMK) (Hajiboland and Ahammed, 2024). The CCaMK pathway is a master regulator that translates the rhizobial signal into a transcriptional reprogramming leading to nodulation. Natural variation in the genes encoding these symbiotic calcium signaling components (e.g., CCaMK, its interacting protein CIPs, and CYCLOPS) influences the efficiency of nodulation and nitrogen fixation across legume species. This direct link between calcium decoding and a key agronomic trait, biological nitrogen fixation, underscores the potential of harnessing natural variation in legume calcium signaling genes not only for abiotic stress tolerance but also for enhanced symbiotic performance and reduced dependency on nitrogen fertilizers.

### 3.3 Mining diversity in wild relatives and landraces

The genetic variation accessible in modern elite cultivars is often limited. Domestication bottlenecks and intensive breeding for yield have inadvertently purged many stress-adaptive alleles that were present in landraces and wild relatives (Singer et al., 2021). Therefore, mining the rich diversity present in crop wild relatives (CWRs) and traditional landraces is essential to overcome this genetic exhaustion (Zhang et al., 2017). Wild relatives, having undergone natural selection, harbor a wide range of favorable alleles for survival. For example, wild barley (*Hordeum* sp*ontaneum*) possesses alleles for longer root length and increased water-use efficiency, while wild rice (*Oryza rufipogon*) carries genotypes with enhanced salt tolerance that are virtually absent in domesticated forms (Zhou et al., 2016; Del Pozo et al., 2024). Landraces, developed over generations in marginal environments, are a critical bridge, containing co-adapted gene complexes that confer resilience under non-ideal conditions (Lazaridi et al., 2024). *De novo* domestication using genome editing represents a promising strategy to rapidly introduce beneficial haplotypes from stress-tolerant wild species into agronomically acceptable backgrounds (Razzaq et al., 2021). Orphan crops, such as finger millet, with their inherent drought tolerance and high calcium accumulation, are also a rich source of unutilized genetic adaptations relevant to calcium signaling (Maharajan et al., 2021). Wild relatives, landraces, and orphan crops represent a significant, underutilised source of genetic diversity crucial for addressing the genetic bottleneck observed in contemporary elite cultivars. The effective use of these resources relies on advanced genomic tools for allele discovery and functional validation, which will be addressed in the subsequent chapter.

## 4 An Integrated toolkit for discovery and validation

The systematic exploitation of natural variation in calcium signaling necessitates an integrated approach that progresses from gene discovery to functional validation and ultimately to application in breeding. This chapter outlines the key technologies that form this pipeline, summarized in **Table 2**. The effective utilization of these resources, however, depends on advanced genomic tools for allele discovery and functional validation, which are the focus of the next chapter.

**Table 2:** Toolkit for exploiting natural variation in calcium signaling networks.

Technology	Primary purpose	Application example (calcium signaling)	Key advantages	Key limitation	Key references
QTL Mapping	Map trait-associated loci (biparental populations).	Identify QTLs for ion homeostasis linked to calcium sensors.	Effective for polygenic traits.	Low resolution, limited diversity.	(Krishnamurthy et al., 2020)
GWAS	Detect SNP-trait associations (diverse panels).	Find SNPs in CPK/CBL/CIPK genes correlated with stress tolerance.	High mapping resolution.	Population structure effects, misses rare alleles.	(Schierenbeck et al., 2023)
Pan-Genomics	Catalog all genes/variations in a species.	Discover presence-absence variation (PAV) in sensor genes missed by a single reference.	Captures full gene repertoire.	Computationally intensive.	(Wicaksono and Buaboocha, 2024)
Transcriptomics (e.g., RNA-seq)	Profile gene expression under defined conditions.	Identify calcium sensor genes upregulated/downregulated by abiotic stress.	Identifies key responsive genes and regulatory networks.	Shows correlation, not necessarily protein-level activity.	(Deepika et al., 2022)
Allele Mining	Identify beneficial alleles in genetic resources.	Discover superior haplotypes in wild relatives and landraces.	Directly links sequence to function.	Requires prior candidate gene knowledge.	(Patra et al., 2021)
CRISPR-Cas9	Precise gene/allele modification.	Validate gene function and introduce beneficial haplotypes.	Establishes causation, precise editing.	Off-target effects, regulatory hurdles.	(Alam et al., 2022)
Genomic Selection (GS)	Predict breeding value (genome-wide markers).	Select for polygenic traits controlled by calcium signaling networks.	Breeds complex traits without knowing causal genes.	Requires large training population.	(Budhlakoti et al., 2022)
HTP Phenotyping	Automated trait measurement (e.g., drones, sensors).	Measure stomatal conductance, canopy temperature related to Ca^2+^ signaling.	High-volume, objective data.	High infrastructure cost.	(Gill et al., 2022)
Multi-Omics Integration	Combine genomic, transcriptomic, proteomic data.	Model how calcium sensor variation affects entire stress-response pathways.	Systems-level view, reveals novel interactions.	High computational complexity, requires specialized expertise.	(Jiang et al., 2025)

This table summarizes the key technologies, their primary applications in calcium signaling research, and their respective advantages and limitations for allele discovery and validation. Key references are provided as representative examples of each technology’s application.

### 4.1 Genomic tools for Identifying functional alleles

The discovery of causal alleles within calcium signaling networks relies on an integrated genomic approach. Traditional quantitative trait locus (QTL) mapping​ in biparental populations identifies large genomic regions associated with stress-induced phenotypes, such as ion homeostasis (Fu and Yang, 2023). However, its resolution is limited. Genome-wide association studies (GWAS)​ leverage historical recombination in diverse germplasm to achieve much finer mapping resolution, enabling the identification of specific SNPs statistically associated with phenotypic variation. GWAS in crops like wheat, barley, and maize have successfully identified SNPs near CDPK, CBL, and CIPK genes linked to drought, salt, and heat-stress responses (Schierenbeck et al., 2023; Slawin et al., 2024).

A fundamental limitation of standard GWAS is its reliance on a single reference genome, which captures only a fraction of a species’ total gene content. This is overcome by pan-genomics, which involves sequencing and assembling multiple genomes from a species to create an inclusive representation of its genomic diversity, including gene presence-absence variation (PAV), copy number variation (CNV), and structural variations (SVs) (Della Coletta et al., 2021). Pan-genome analyses in soybean and tomato have revealed that the reference genome misses complete stress-signaling genes present in wild accessions, highlighting an untapped reservoir of resilience alleles (Bayer et al., 2020; Ni et al., 2023). Genomic tools effectively identify candidate alleles linked to stress tolerance; however, establishing a causal relationship necessitates transitioning from correlation to causation, a process for which precision genome editing is particularly well-suited.

### 4.2 Functional validation via genome editing

The final step in transitioning from correlation to causation is functional validation. CRISPR-Cas9-mediated genome editing is a pivotal tool for this, allowing for the precise knockouts of genes, the replacement of alleles, or the targeted introduction of beneficial haplotypes. For example, CRISPR-Cas9 has been used to demonstrate that introducing a favorable CIPK haplotype from a tolerant variety into a sensitive one enhances ion balance under salt stress (Alam et al., 2022). In *Arabidopsis*, targeted mutagenesis of genes like OST2 has validated their role in stomatal closure and water-use efficiency (Ruggiero et al., 2017). This technology enables the functional assessment of natural alleles in an otherwise uniform genetic background, controlling for epistatic effects. It also facilitates the stacking of multiple favorable alleles to engineer polygenic traits, such as drought tolerance, moving beyond single-gene analyses (Chen et al., 2024). The ability to precisely validate gene function and engineer beneficial haplotypes with genome editing marks a paradigm shift. The subsequent challenge is to integrate these validated findings into practical breeding frameworks that can deliver improved varieties to farmers.

### 4.3 Breeding strategies: from MAS to genomic selection

The ultimate goal of allele discovery is its application in breeding. Marker-assisted selection (MAS)​ uses linked markers to introgress major-effect QTLs into elite lines, as demonstrated by the introgression of the SaltolQTL for salinity tolerance in rice (Krishnamurthy et al., 2020). However, MAS is inefficient for polygenic traits. Genomic selection (GS) overcomes this limitation by utilizing genome-wide marker information to predict the breeding value of individuals for complex traits, thereby enabling selection for polygenic attributes controlled by calcium signaling networks (Budhlakoti et al., 2022). The integration of these molecular approaches with high-throughput phenotyping (HTP), utilizing drones and automated systems to measure traits such as stomatal conductance and canopy temperature, enables the large-scale screening of breeding populations under field conditions (Gill et al., 2022). The process of advancing from allele discovery to crop enhancement depends on a cohesive framework: genomic tools for identification, genome editing for validation, and sophisticated breeding strategies such as GS for introgression. The effective use of this toolkit depends on addressing substantial translational gaps between controlled laboratory settings and intricate field conditions.

## 5 Research gaps and a framework for climate-resilient crops

### 5.1 Bridging the lab-to-field validation gap

A significant challenge in translating calcium signaling research into agricultural application is the lack of validation under real-world field conditions. While the roles of many calcium sensors have been elucidated in controlled environments, their contributions to yield stability and resilience under complex, variable field conditions remain largely speculative (Ghosh et al., 2022). For instance, the anticipated yield benefit of enhancing SOS pathway genes in rice under salt stress lacks confirmation from large-scale, multi-location field trials across diverse saline environments (Liu et al., 2022a). Similarly, the promise of single-gene editing often faces challenges due to epistatic interactions and genotype-by-environment (G × E) effects that are only apparent in the field (Baier et al., 2023). Therefore, a major future direction must be the implementation of large-scale field phenotyping to directly quantify the impact of calcium sensor alleles on crop productivity and stability. The primary translational challenge is the absence of empirical data from field trials, which is essential for validating the agronomic value of calcium sensor alleles identified in controlled environments.

### 5.2 Understanding calcium signaling under combined abiotic and abiotic-biotic stresses

In natural agricultural settings, crops typically endure multiple concurrent abiotic stresses (e.g., drought and heat) or combinations of abiotic and biotic stresses (e.g., salinity, which weakens plant defenses against pathogens). Most calcium signalling studies primarily examine responses to individual stressors. The relationship between calcium signalling pathways in these complex scenarios remains inadequately understood, frequently characterised by antagonistic crosstalk and trade-offs (Radha et al., 2023; Jiang et al., 2025). For example, CDPK-mediated ABA signaling, which confers drought tolerance by closing stomata, may simultaneously reduce heat tolerance by limiting transpirational cooling and increase susceptibility to pathogens by repressing salicylic acid-dependent defense pathways (Kaya et al., 2024). Calcium signals are crucial in coordinating responses to combined stresses, particularly in the intricate relationship between drought and pathogen response signalling, as reviewed by Patra et al. (2021). Consequently, a major research frontier lies in deciphering the often antagonistic crosstalk between calcium-dependent pathways under combined stresses, which is critical for avoiding unintended trade-offs in breeding programs.

### 5.3 Expanding the search for novel variation

**Figure 4:**
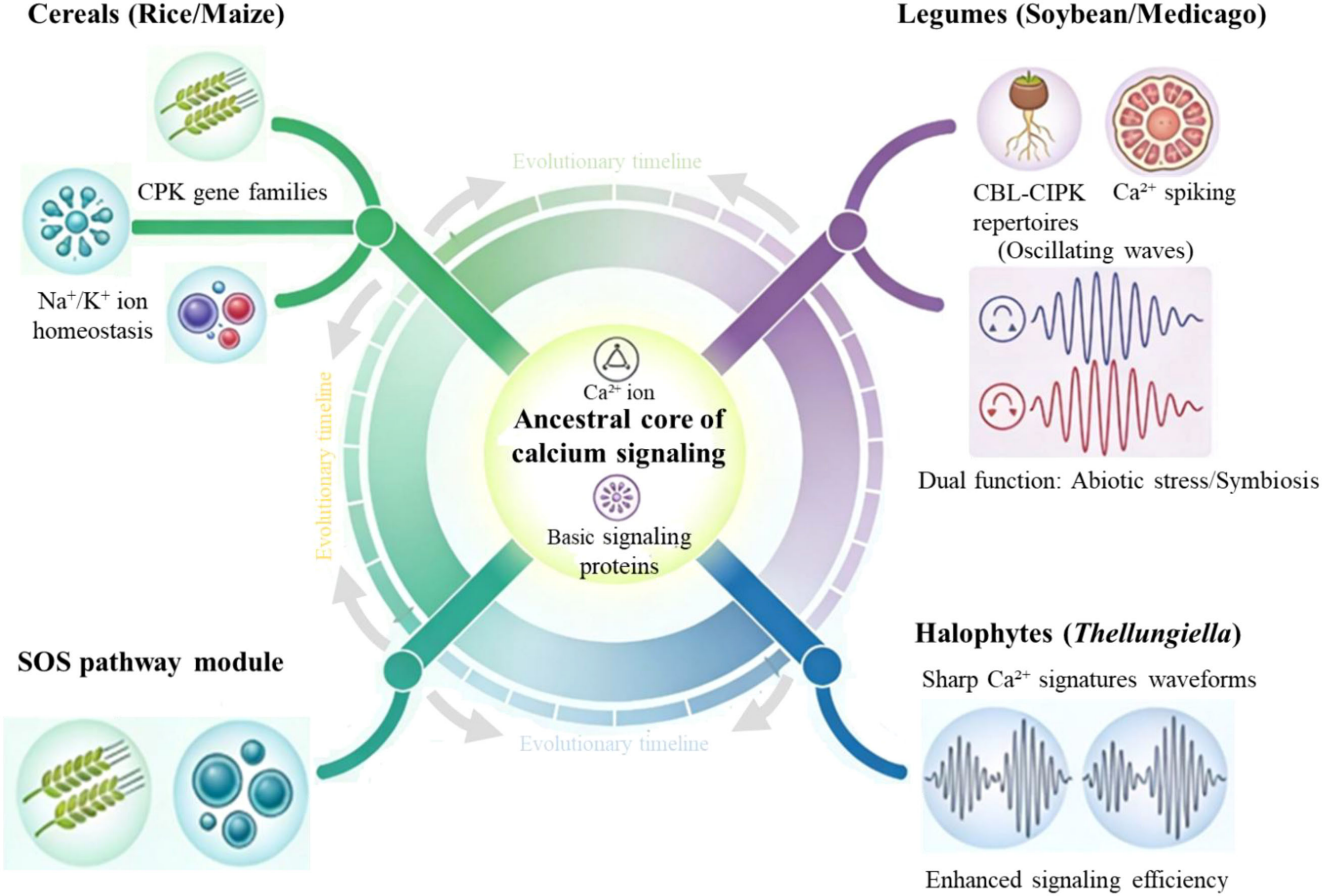
Evolutionary diversification and functional specialization in major crops. Evolutionary diversification of the ancestral Ca^2+^-signaling core across plant lineages. The central circle depicts a conserved ancestral core of Ca^2+^-mediated signaling composed of basic signaling proteins that emerged early in land plant evolution. From this core, distinct lineage-specific modules have radiated along the evolutionary timeline, including CPK gene families in cereals (e.g., rice and maize) that regulate Na^+^/K^+^ ion homeostasis and the SOS pathway module associated with salinity tolerance. In legumes (e.g., soybean and medicago), expanded CBL–CIPK repertoires and specialized Ca^2+^-spiking oscillations underpin dual functions in abiotic stress adaptation and symbiotic interactions, whereas in halophytes such as *Thellungiella*, sharp Ca^2+^-signatures waveforms are proposed to confer enhanced signaling efficiency under extreme salinity.

While significant progress has been made in model species and major cereals, the full potential of genetic​ variation in calcium signaling remains underexplored. Future research should broaden its scope to encompass orphan crops, such as finger millet and teff, as well as legumes, including chickpea, soybean, and common bean, in addition to extreme halophytes and xerophytes. These species have developed under continuous stress and may possess unique, resilient alleles (Jamra et al., 2024; Dinakarkumar et al., 2025). The evolutionary diversification and functional specialization of calcium signaling components across these and major crops, as depicted in **Figure 4**, provide a compelling rationale for mining these underexplored genetic resources. Deeper mining of crop wild relatives (CWRs) through pan-genomic analyses will also be essential to uncover presence-absence variations and rare alleles missing from elite gene pools. Furthermore, integrating high-throughput phenotyping (HTP) with advanced genomics, utilizing drones, sensors, and AI-driven image analysis, will be crucial for capturing complex physiological traits and linking them to genetic​ variation in calcium signaling genes under field conditions (Gill et al., 2022). Thus, unlocking the full potential of calcium signaling for crop improvement requires a deliberate expansion of search efforts to underexplored genetic resources, coupled with advanced phenomics to capture complex traits.

### 5.4 The interplay of calcium signaling and the epigenetic landscape

The interaction between calcium signalling and the epigenetic landscape of plants represents a significant and developing area of research. Genetic variation in coding sequences is important; however, the expression of calcium sensors and their downstream targets is additionally affected by epigenetic modifications, such as DNA methylation and histone modifications. These modifications can be dynamically adjusted in response to environmental stresses, potentially regulated by Ca^2+^ signals, resulting in stable “epialleles.” This indicates a layer of variation that is not contingent upon alterations in DNA sequence. A recent study on tomatoes indicated that drought-induced Ca^2+^ spikes are associated with specific histone modifications that regulate the expression of stress-responsive genes, facilitating acclimation (Ge et al., 2025). Comprehending this interaction is crucial, as it may elucidate the mechanism by which transient Ca^2+^ signals contribute to long-term transcriptional memory and potentially facilitate transgenerational inheritance of stress resilience. Future research should extend to investigate the epigenomic aspects of calcium signalling. Essential enquiries involve the mechanisms by which distinct Ca^2+^ signatures attract chromatin remodelers. What is the extent of natural epiallelic variation in the regulatory regions of genes such as CPKs and CBL-CIPKs in contributing to stress resilience? Investigating the epigenetic dimension is essential for a comprehensive understanding of signalling regulation and for its application in breeding, potentially via epi-breeding strategies that favour advantageous epigenetic states.

### 5.5 Transitioning to a crop-centric research paradigm

The field’s heavy reliance on *Arabidopsis thaliana* as a model organism, while foundational, presents a translational bottleneck. Arabidopsis is a salt-sensitive dicot with a genetic architecture and stress responses that often differ significantly from major monocot crops (Uauy et al., 2025). Future research must prioritize crop-centric studies, focusing on functional validation of alleles directly in agronomically important species like cereals, legumes, and oilseeds using tools like CRISPR-Cas9 (Ndudzo et al., 2024). In practical terms, accelerating translational impact necessitates a strategic reallocation of research focus toward functional studies directly in key crops, moving beyond the limitations of the Arabidopsis model.

### 5.6 Synthesis and a framework for future research

**Figure 5:**
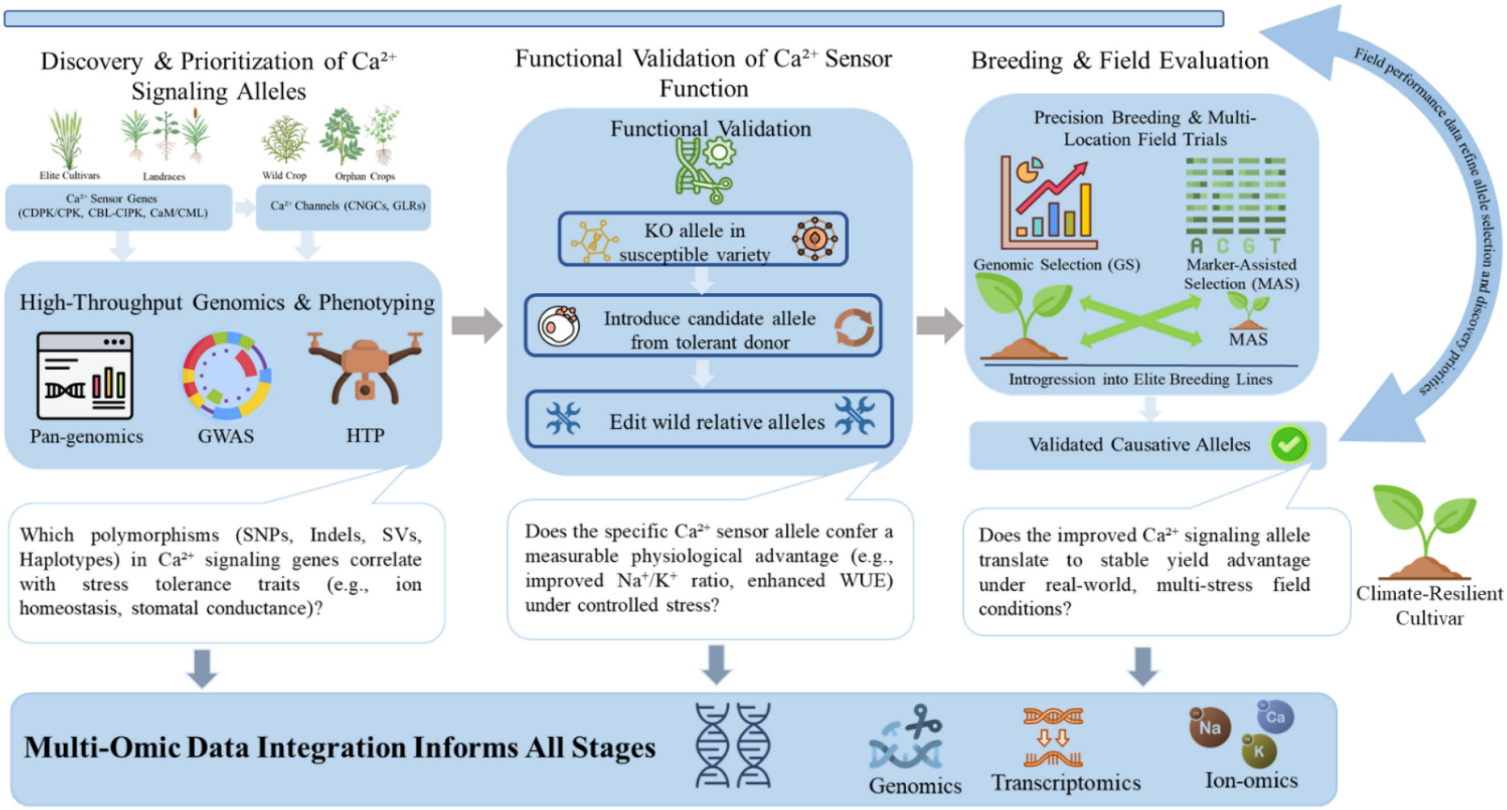
An integrated pipeline for harnessing natural variation in calcium signaling to develop climate-resilient crops.

In conclusion, while significant molecular insights into calcium signaling have been achieved, the path to leveraging this knowledge for crop improvement requires addressing key gaps. Future research must be guided by a framework that prioritizes the integration of genomic discovery, functional validation, and breeding application, as outlined in the translational pipeline in **Figure 5**. This integrated approach, which moves from allele discovery in diverse germplasm to field validation in target crops, is essential for developing the next generation of climate-resilient varieties.

## 6 Conclusion

Calcium signalling is a crucial element in plant environmental perception, and the inherent genetic diversity within its sensor networks constitutes a significant, yet largely untapped resource for enhancing crop performance. Domestication and intensive breeding have led to a genetic bottleneck, eliminating adaptive alleles from wild relatives and landraces that are essential for resilience. This review emphasises the potential of high-resolution genomics and precision genome editing to mitigate this erosion.

Identifying advantageous haplotypes within the CBL-CIPK and CDPK families and utilizing CRISPR-Cas9 for their deployment provides a straightforward approach to improving tolerance to complex abiotic stresses. Bridging the translational gap from the laboratory to the field necessitates the integration of genomic selection, multi-omics approaches, and the strategic utilization of resilient wild species. The integration of new insights into epigenetic regulation and complex phytohormone feedback loops enhances the framework for manipulating these networks.

Harnessing the natural diversity of the calcium signalling toolkit is essential for the development of climate-resilient crops that can protect global food systems in a changing climate. Integrating mechanistic discovery with applied breeding via translational genomics can expedite the creation of crops capable of withstanding the abiotic stresses that pose growing challenges to global agriculture.

## Glossary

ABA: Abscisic Acid
CBF: C-repeat Binding Factor
CNGCs: Cyclic Nucleotide-Gated Cation Channels
COR: Cold-Regulated (genes/proteins)
GLR: Glutamate Receptor-Like
HKT: High-Affinity K^+^ Transporter
HTP/HTPP: High-Throughput Phenotyping
MAPK/MAPKs: Mitogen-Activated Protein Kinase(s)
MAS: Marker-Assisted Selection
RLKs: Receptor-Like Kinases
SNP/SNPs: Single Nucleotide Polymorphism(s)
SOS: Salt Overly Sensitive (Pathway)
CaM/CaMs: Calmodulin(s)
CBL/CBLs: Calcineurin B-Like Protein(s)
CDPKs/CPKs: Calcium-Dependent Protein Kinase(s)
CIPK/CIPKs: CBL-Interacting Protein Kinase(s)
CML/CMLs: Calmodulin-Like Protein(s)
